# Identification of Natural-Product Inhibitors of the 2*C*‑Methyl‑d‑erythritol 4‑Phosphate Pathway

**DOI:** 10.1021/acsmedchemlett.6c00054

**Published:** 2026-03-13

**Authors:** Eleonora Diamanti, Alaa Alhayek, Antoine Lacour, Mohammad Walid Shahrour, Daan Willocx, Boris Illarionov, Markus Fischer, Marc Stadler, Norbert Reiling, Jennifer Herrmann, Rolf Müller, Anna K. H. Hirsch

**Affiliations:** † 443745 Helmholtz Institute for Pharmaceutical Research (HIPS)|Helmholtz Centre for Infection Research (HZI), 66123 Saarbrücken, Saarland, Germany; ‡ Saarland University, Department of Pharmacy, Campus Building E8.1, 66123 Saarbrücken, Saarland, Germany; § PharmaScienceHub, Campus E2.1, 66123 Saarbrücken, Saarland, Germany; ∥ Hamburg School of Food Science, 14915 Institute of Food Chemistry, Grindelallee 117, 20146 Hamburg, Germany; ⊥ Department of Microbial Drugs, Helmholtz Centre for Infection Research (HZI), 38124 Braunschweig, Germany; # Microbial Interface Biology, 28413 Research Center Borstel, Leibniz Lung Center, Parkallee 1 Borstel 23845, Germany; a German Centre for Infection Research (DZIF), Partner Site Hannover-Braunschweig, 38124 Braunschweig, Germany; b German Center for Infection Research (DZIF), Partner Site Hamburg-Lübeck-Borstel-Riems, Borstel 23845, Germany

**Keywords:** MEP pathway, natural product, DXPS, IspD, maracen A, polyketomycin

## Abstract

To tackle the emerging resistance against existing antibiotics, we screened natural-product (NP) libraries against two underexploited target enzymes from the 2*C*-methyl-d-erythritol 4-phosphate (MEP) pathway, namely, *Mycobacterium tuberculosis* DXPS and *Escherichia coli* IspD. We have chosen these two enzymes due to the availability of the crystal structures that helped to elucidate the putative binding modes of the NPs identified. The screening of a NP collection led to the discovery of myxobacteria-derived maracen A and Streptomyces-derived polyketomycin as the first NPs targeting these enzymes.

## Graphical abstract

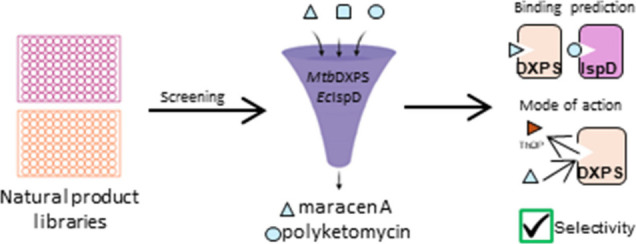

## Introduction

There is an increasingly compelling case for reconsidering the use of natural products (NPs) for anti-infective or antibiotic drug discovery. Over the past 40 years, approximately 60% of all new antibacterial agents have been NPs or derivatives of them.^1,2^ The global antibacterial clinical pipeline, however is mainly populated by modifications of existing antibiotic classes, requiring it to be constantly fed and strengthened with new entities to meet clinical needs.^3^ This is even more urgent due to the ever-increasing prevalence of resistance, which requires the discovery and development of new chemotypes against new biological targets.

Here, we combined the use of NP libraries with an untapped biological source, the 2*C*-methyl-d-erythritol 4-phosphate (MEP) pathway, to identify NPs endowed with novel modes of action.^4^ The MEP pathway that is absent in humans but essential for medically relevant pathogens (e.g., *Plasmodium falciparum*, *Mycobacterium tuberculosis*, and *Klebsiella pneumoniae* as a representative Gram-negative bacterium) represents an attractive source of anti-infective targets. The MEP pathway includes seven enzymes that are responsible for the biosynthesis of isoprenoid isopentenyl diphosphate (IDP) and its isomer dimethylallyl diphosphate (DMADP). Both IDP and DMADP are universal precursor for the biosynthesis of secondary metabolites essential for bacterial growth and survival (Figure 1).^5^ Fosmidomycin, now in clinical trials to treat malaria, is an NP isolated from *Streptomyces lavendulae* that targets the IspC enzyme, further validating MEP-pathway enzymes as attractive drug targets for anti-infective development.^6^

**Figure 1:**
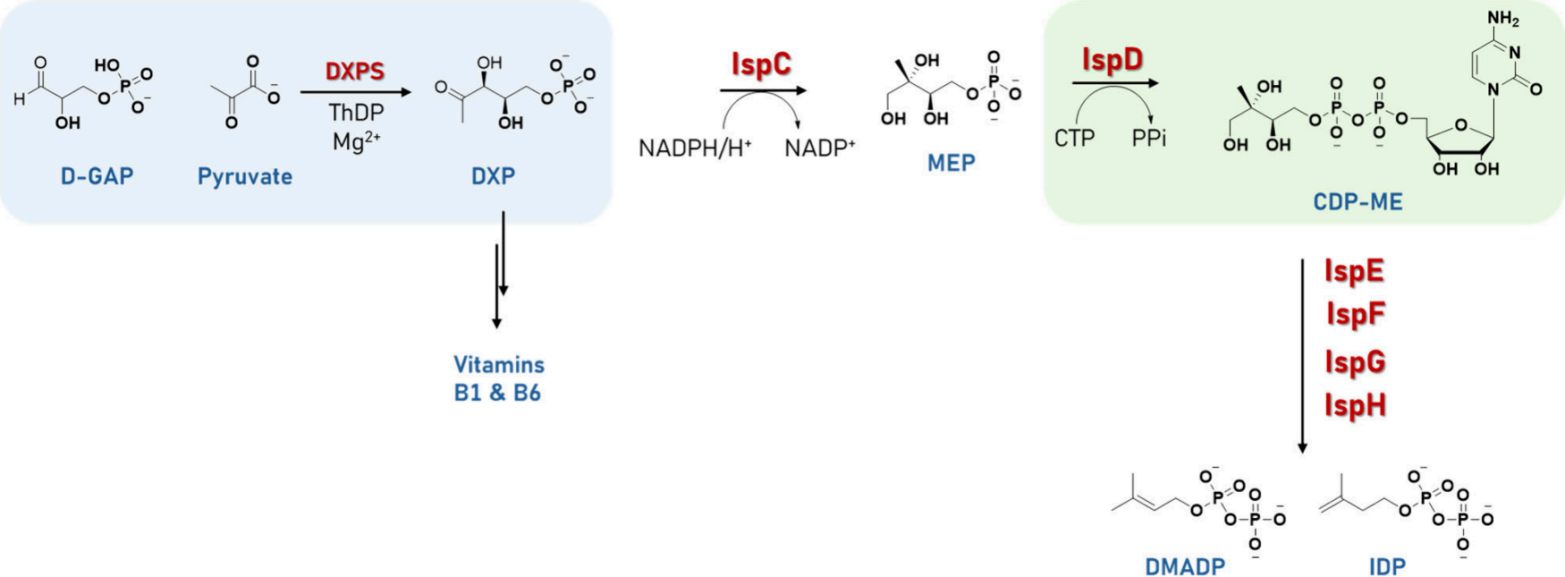
2*C*-Methyl-d-erythritol 4-phosphate (MEP) pathway with the enzymes studied in this work (DXPS and IspD) that are highlighted in the boxes. 1-Deoxy-d-xylulose 5-phosphate synthase (DXPS); 1-deoxy-d-xylulose-5-phosphate reductoisomerase (IspC); 4-diphosphocytidyl-2*C*-methyl-d-erythritol cytidylyltransferase (IspD); 4-diphosphocytidyl-2*C-*-methyl-d-erythritol kinase (IspE); 2*C*-methyl-d-erythritol-2,4-cyclodiphosphate synthase (IspF); 1-hydroxy-2-methyl-2-(*E*)-butenyl-4-diphosphate synthase (IspG); 4-hydroxy-3-methyl-2-(*E*)-butenyl-4-diphosphate reductase (IspH).

In this study, we focused on both *M. tuberculosis* 1-deoxy-d-xylulose 5-phosphate synthase DXPS (*Mt*DXPS) and *Escherichia coli* IspD (*Ec*IspD). DXPS, the first enzyme in the MEP pathway, is a thiamine diphosphate (ThDP)-dependent enzyme involved in the catalytic decarboxylative condensation of pyruvate and d-glyceraldehyde 3-phosphate (d-GAP) in a thiamine diphosphate (ThDP)-dependent manner to produce a branch point product called DXP. The latter one is involved in the biosynthesis of vitamins B–1 and B–6, besides the production of the isoprenoid building blocks (Figure 1).^7^ Inhibiting DXPS disrupts the flux of metabolites into the pathway, making the enzyme a promising antibiotic target, while IspD, the third enzyme in the MEP pathway, catalyzes the formation of 4-diphosphocytidyl-2-*C*-methylerythritol (CDP-ME) from MEP and cytidine triphosphate (CTP) (Figure 1).^8^

For this study, we selected *Mt*DXPS and *Ec*IspD as the available crystal structures to provide valuable insights into possible binding mode of inhibitor. As a note, for *Mt*DXPS we succeeded in determining the crystal structure of the *holo* protein of Δ*Mt*DXPS with a resolution of 1.85 Å (PDB ID: 7A9H),^9^ while for *E. coli* IspD we used the high-resolution structures of *E. coli* CDP-ME synthetase in the *apo* form and complexed them with both CTP–Mg^2+^ and CDP-ME–Mg^2+^.^10^

Our stepwise workflow based on a biochemical enzyme activity assay led to the identification of two novel NP hit compounds that selectively target *Mt*DXPS and *Ec*IspD.

## Results and Discussion

### NP Library

NPs in comparison to conventional synthetic molecules, possess unique properties that offer both advantages and challenges to the drug-discovery process. For example, they are characterized by a high level of scaffold diversity, as well as structural complexity. Their most typical peculiarities compared with synthetic compound libraries are higher molecular mass, more stereogenic centers, a larger fraction of sp^3^-hybridized carbon atoms, more oxygen atoms but fewer nitrogen and halogen atoms, higher numbers of solvated hydrogen bond donors and acceptors, and more molecular rigidity.^11^ Challenges are, in general, complex chemistry, low compound yields, toxicity, stability issues, and difficulties in large-scale production.^12^ Here, we took advantage of NP libraries to identify novel hits against the enzymes *Mt*DXPS and *Ec*IspD. Specifically, for the *Mt*DXPS screening we used a total of 259 purified NPs derived from myxobacteria and 88 from fungi that were part of the DZIF (German Center for Infection Research) NP libraries (https://www.dzif.de/en/novel-antibiotics), while for *Ec*IspD the screening library consisted of 478 compounds obtained from AnalytiCon Discovery (Potsdam, Germany), 210 compounds from ASINEX (Winston-Salem, NC, USA) (Class II), 20 nucleic acid building blocks, 174 NPs from BioViotica (BioVL), and 259 myxobacterial secondary metabolites from the DZIF collection.

### *M. tuberculosis* DXPS (*Mt*DXPS) Screening

The first step in our hit-identification strategy involved screening at 50 μM of the myxobacterial (259 compounds) and fungal (88 compounds) collections (Figure S1). This screening resulted in 16 hits, 9 of which were derived from myxobacteria and 7 from fungal sources (inhibition level >40%, Table S1). From the pure compound hits, we excluded some due to known stability issues (*e.g.*, sulfangolids), and some others were not available on a larger scale. In turn, confirmatory screening was continued with six pure NPs (maracen A, sorangiolid A, truncaton A, obionin A, obionin C, and hexadecanoic acid) for which we saw the highest chances for continuation into hit validation. The selected compounds were subjected to a counter screening where we checked for possible compound interference with the fluorescent readout, leading to false positives (Figure S2). As truncaton A and obionin C reduced the signal of NADPH rather than inhibiting the activity of the enzyme, they have been excluded from our hit-selection process. This was not observed for other hits including maracen A, hexadecanoic acid, sorangiolid A, and obionin A. The next step was the determination of the IC_50_ values (Figure S3), where butylacetylphosphonate (BAP),^13^ a known DXPS inhibitor, has been included as a positive control. The IC_50_ values revealed that maracen A was equipotent to BAP with an IC_50_ value of 10.3 ± 0.02 μM; sorangiolid A has an IC_50_ value of 21.2 ± 0.2 μM; and moderate activities with IC_50_ values of 72.2 ± 6.4 and 55.2 ± 3.3 μM have been found for hexadecanoic acid and obionin A, respectively. Based on these results, we selected maracen A for further study, and the next step was to test the selectivity of our hit over the mammalian pyruvate dehydrogenase (PDH) enzyme since it has a very similar tertiary structure to that of DXPS, although it shares only 23% sequence identity with DXPS.^13^ We were pleased to see no inhibition of PDH, indicating that maracen A is a selective inhibitor of *Mt*DXPS over other mammalian ThDP-dependent enzymes (Figure S4). Due to the flexible structure of the NP with the aim of eliminating unspecific binding, we tested maracen A activity against other MEP enzymes (Table S2). We found no activity against PDH, PK-LDH, *Ec*IspD, and *Ec*IspE and an IC_50_ of 26 ± 7 μM against *Pf*IspD.

### Maracen A Competes with ThDP at the Active Site

Considering that maracen A has an IC_50_ value comparable to BAP, we further investigated whether maracen A is a ThDP-competitive inhibitor and conducted a competition study including both of them in the assay. Maracen A was titrated in the presence of four different concentrations of ThDP, which were altered according to the *K*_M_ value obtained (Table S3). With increasing concentrations of ThDP, the IC_50_ values for maracen A increased in parallel. At a concentration of 1× *K*_M_ of ThDP (0.3 μM), maracen A showed an IC_50_ value of 11.1 ± 1.9 μM, whereas the IC_50_ values were 26.3 ± 6.4 μM and 92.6 ± 37.8 μM at 10× *K*_M_ (*i.e.*, 3 μM) and 100× *K*_M_ (*i.e.*, 30 μM), respectively (Figure 2). These findings clearly show that maracen A competes with ThDP at the active site.

**Figure 2:**
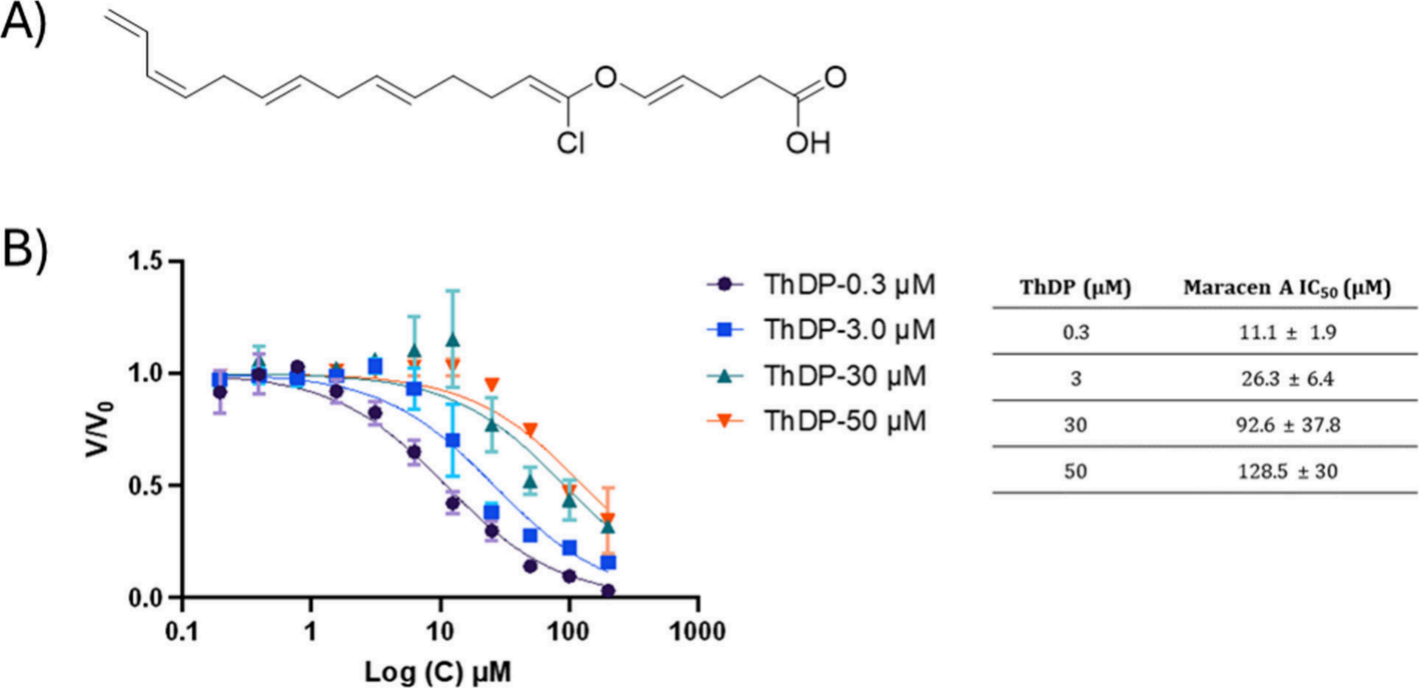
(A) Chemical structure of maracen A. (B) Plot illustrating the competition experiment between Thiamine diphosphate (ThDP) and maracen A. The determined IC_50_ value of maracen A at various ThDP concentrations using the coupled enzymatic assay (DXPS-IspC). *Mt*DXPS: *Mycobacterium tuberculosis* 1-deoxy-d-xylulose 5-phosphate synthase. Means ± SD of two independent experiments are shown.

The absence of an IC_50_ shift for maracen A at 1× *K*_M_ ThDP may reflect a near-equimolar balance between the inhibitor and cofactor or suggest a nonclassical or mixed mode of inhibition rather than competition alone. Notably, BAP, which forms a covalent phosphonolactyl–ThDP (PL-ThDP) adduct and, thereby, traps the cofactor in an inactive state shows a similar IC_50_ to maracen A at 1× *K*_M_, suggesting that alternative mechanisms may also contribute. These include slow-binding kinetics, mixed-mode inhibition, or a ThDP-dependent binding mode that does not involve only direct competition for the cofactor binding site.

To further explore the putative binding mode, we performed molecular docking of maracen A against *Mt*DXS using the crystal structure of *Mt*DXPS with ThDP (PDB code: 7A9H).^9^ As attempts to obtain a cocrystal structure failed, we performed docking studies retaining the Mg^2+^ atom and two important Mg-coordinating water molecules (HOH857 and HOH959) (Figure 3). We chose this methodological approach due to the presence of a potential Mg-coordinating functional group (carboxylic acid) in maracen A, thus warranting the preservation of the aforementioned conserved water molecules. Indeed, maracen A was predicted to bind to the Mg^2+^ atom within the *Mt*DXPS catalytic site via an interaction with the carboxyl group that was also predicted bind to His99 effectively, forming a bridge between His99 and the Mg^2+^ atom. This is reminiscent of the binding mode of ThDP (PDB code: 7A9H) where the phosphate moiety also forms a bridge between His99 and the Mg^2+^ atom (along with coordinating 2 additional waters). An additional interaction between the ether oxygen atom of maracen A and Lys207 was observed. Tight coordination of the Mg^2+^ cation by two water molecules, Asp172 and Asn201, was observed with Asn199 and Asp172 also coordinating both water molecules. The hydrophobic tail of maracen A was found to partially occupy the rest of the ThDP binding site, forming hydrophobic interactions with Phe380, Ile353, and Met331. Overall, the carboxyl group binding mode is reminiscent of the binding of the phosphate moiety of ThDP. These findings suggest that maracen A binds to the ThDP site in a ThDP-sensitive but not a strictly competitive fashion, consistent with the IC_50_ shift data. While molecular docking supports overlapping interactions with the ThDP phosphate binding region, the lack of IC_50_ shift at 1× *K*_M_ may reflect a mixed or partially overlapping mode of inhibition. Together, the structural and enzymatic data indicate that maracen A may interfere with ThDP-dependent catalysis through a nonclassical mechanism.

**Figure 3:**
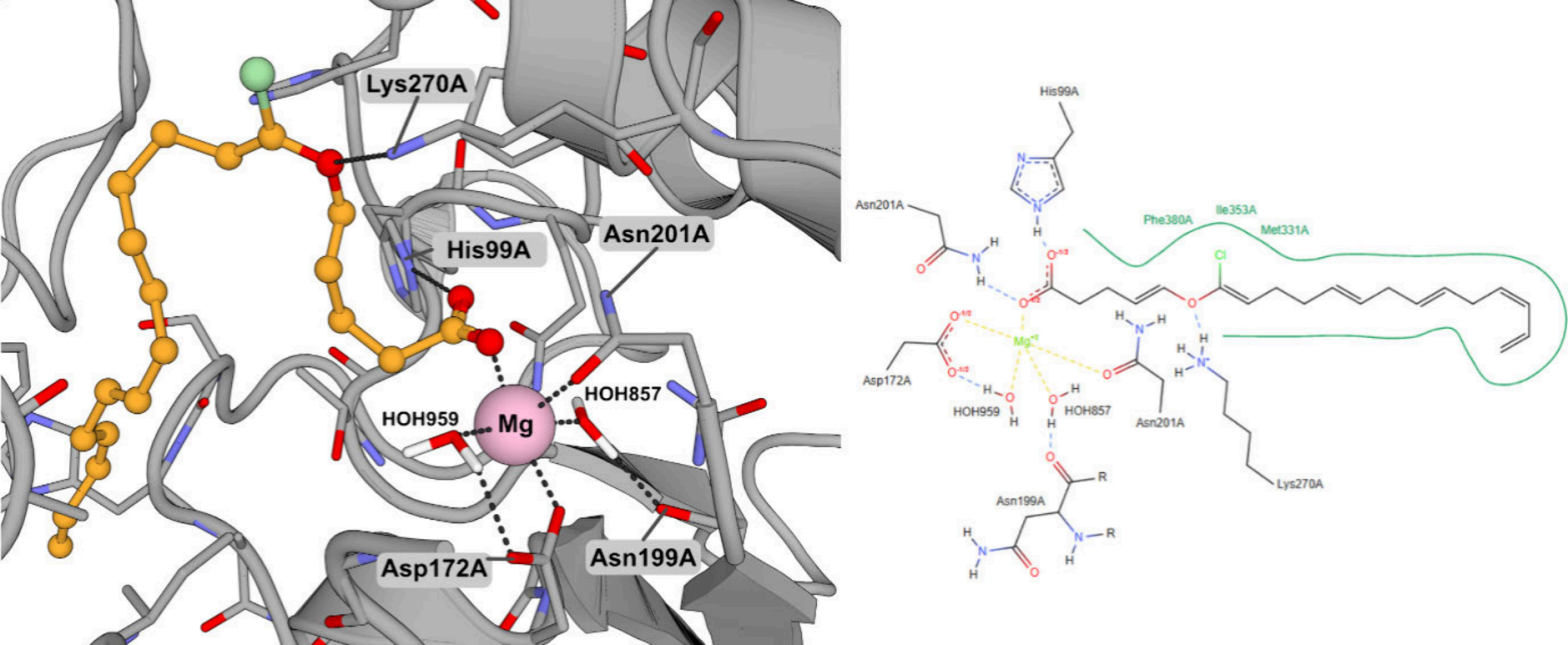
(left) 3D representation of the predicted binding mode of maracen A to *Mycobacterium tuberculosis* 1-deoxy-d-xylulose 5-phosphate synthase (*Mt*DXPS). Maracen A is shown in orange, and the magnesium atom is shown in pink. Interacting residues are labeled, and polar interactions are shown as black, dashed lines. (right) 2D interaction diagram of maracen A with *Mt*DXPS.

Previous reports have suggested an antitubercular effect of maracen A; however, the authors did not provide experimental details regarding assay conditions, target specificity, or mechanism of inhibition.^14^ We therefore re-evaluated a putative *Mtb* whole-cell activity of maracen A, and it did not show reproducible antitubercular activity in our tests. This is in line with independent observations since a rescreening of myxobacterial NPs with putative anti-Mtb properties did not identify maracen A as a hit in an MGIT-based assay (unpublished data). Our observation is that maracen A shows an IC_50_ of 10.3 μM in *Mt*DXPS enzymatic assays but lacks activity in whole-cell activity assays, indicating that maracen A may not reach its target within the bacteria. The underlying mechanisms are currently not clear and needs further investigation.

### *E. coli* IspD (*Ec*IspD) Screening

The enzymatic activity of the IspD enzyme was measured in a photometric assay, coupling *Ec*IspD activity to the chromogenic reaction of NADH oxidation to allow near-UV photometric monitoring. Specifically, IspD is responsible for catalyzing the conversion of MEP and CTP to CDP-ME. The latter is one of the substrates of the IspE enzyme, which uses ATP as a second substrate and the ADP released by IspE to trigger a cascade of events catalyzed by pyruvate kinase (PK) and lactate dehydrogenase (LDH), leading to NADH oxidation, which is detectable by photometry at 340 nm (Scheme S1). Therefore, following this assay, the compounds were also checked for inhibition against *Ec*IspD, *Ec*IspE, and PK-LDH.^15^ Checking three different enzymes might help to reduce the risk of nonspecific interference with the assay and avoid the risk that hit compounds coming from the IspD enzyme-based approach may fall under the pan-assay interference (PAIN) subcluster.

On this basis, the first step was to measure all the compounds at a concentration of 25 μM which led to the selection of 15 hits (Table S4). Then, we focused on the top-8 hit compounds: isochaetochromin B1, palmarumycin, pentabromopseudilin, polyketomycin, simocyclinone D4, skyrin, and truncatons A and C that showed a percentage of inhibition higher than 55 at the tested concentration.

We then excluded truncatons A and C as they could not be isolated in a pure form needed for further study and, additionally, isochaetochromin B1, which has been already reported to act as an inhibitor of triacylglycerol synthesis in mammalian cells.^16^

We restricted our screening for IC_50_ determination to skyrin, palmarumycin, simocyclinone D4, polyketomycin, and pentabromopseudilin. From the data reported in Section S7.1, we excluded simocyclinone D4 from further studies due to its moderate activity (*Ec*IspD IC_50_ = 99 ± 12 μM). We were pleased to see that our unbiased HTS selected pentabromopseudilin as an IspD inhibitor (*Ec*IspD IC_50_ = 36 ± 6 μM); these data demonstrate robustness of selection, as this chemical class has already been reported as an IspD inhibitor for the plant homologue IspD from *Arabidopsis thaliana* (*At*IspD) and IspD from *Plasmodium vivax* (*Pv*IspD).^17^

As previously mentioned, considering that we are using a coupled assay, we also checked the activity of our hits against auxiliary enzymes *Ec*IspE and PK-LDH (Table S5 and Figure 2). By doing so, we confirmed that the most active hits, in fact, selectively inhibit the target enzyme and not the auxiliary enzymes of the IspD assay. The IC_50_ evaluation against PK-LDH led to the exclusion of skyrin and pentabromopseudilin as selective IspD inhibitors as they showed an IC_50_ of 73 ± 10 μM and 30 ± 4 μM, respectively. Furthermore, a recent study showed that the antibacterial activity of pseudilins may be due to their protonophoric activity.^18^ In summary, we conclude that palmarumycin and polyketomycin behave as selective IspD inhibitors due to the absence of activity against *Ec*IspE and PK-LDH.

Nevertheless, we decided in our next step to check the cellular activity of all four hits against a small panel of Gram-positive and -negative bacteria as well as their toxicity in the KB3.1 cell line (Table S6).

We were pleased to find that polyketomycin in the presence of the outer-membrane permeabilizer polymyxin B nonapeptide (PMBN)^19^ is able to inhibit growth of *E. coli* (MIC = 4 μg/mL), while it was inactive (MIC > 128 μg/mL) against several *E. coli* strains, including efflux-deficient mutants, under normal growth conditions. This points to a permeability issue in Gram-negative bacteria. However, it should be mentioned that the potent activity against Gram-positive bacteria (MIC = 0.06 μg/mL against *Enterococcus faecium* and *Staphylococcus aureus*) and moderate cytotoxicity (KB3.1 IC_50_ = 19.2 μg/mL), along with previous reports on various biological activities, suggest that cellular activity of polyketomycin is not solely driven by inhibition of IspD.^20^ Regarding palmarumycin, it was excluded from further study as it did not show any promising cellular activities (Table S6).

In summary, we moved on with polyketomycin, a tetracyclic quinone glycoside isolated from *Streptomyces diastatochromogenes* Tü 6028.^21^ Chemically, it consists of two polyketide moieties: a decaketide-derived polyketomycinone and a tetraketide-derived 3,6-dimethylsalicylic acid.^22^ It has been reported to exhibit antibacterial, cytotoxic, and antiplasmodial activity, but its mechanism of action has not been defined yet. Therefore, we were interested to further check its putative binding mode, and we performed molecular docking to understand how polyketomycin might interact with *Ec*IspD.

To do so, we used the crystal structure of *Ec*IspD with CTP (PDB code: 1I52) given the better resolution and completeness of the catalytic site when compared to the *apo* (PDB code: 1INJ) and CDP-ME-bound structures (PDB code: 1INI).^10^ A previous study defined the binding site based on CDP-ME rather than CTP.^23^ We elected to follow a similar strategy to both include the putative MEP binding site and accommodate the large size of polyketomycin.

Polyketomycin was predicted to form several interactions including hydrogen bonds to the side chains of Lys213 and Thr189 as well as hydrophobic interactions with Gly16 and Arg85 (Figure 4). The predicted binding mode partially occupies the CTP binding pocket as well as the putative MEP binding pocket, which could explain the inhibitory activity of polyketomycin against *Ec*IspD.

**Figure 4:**
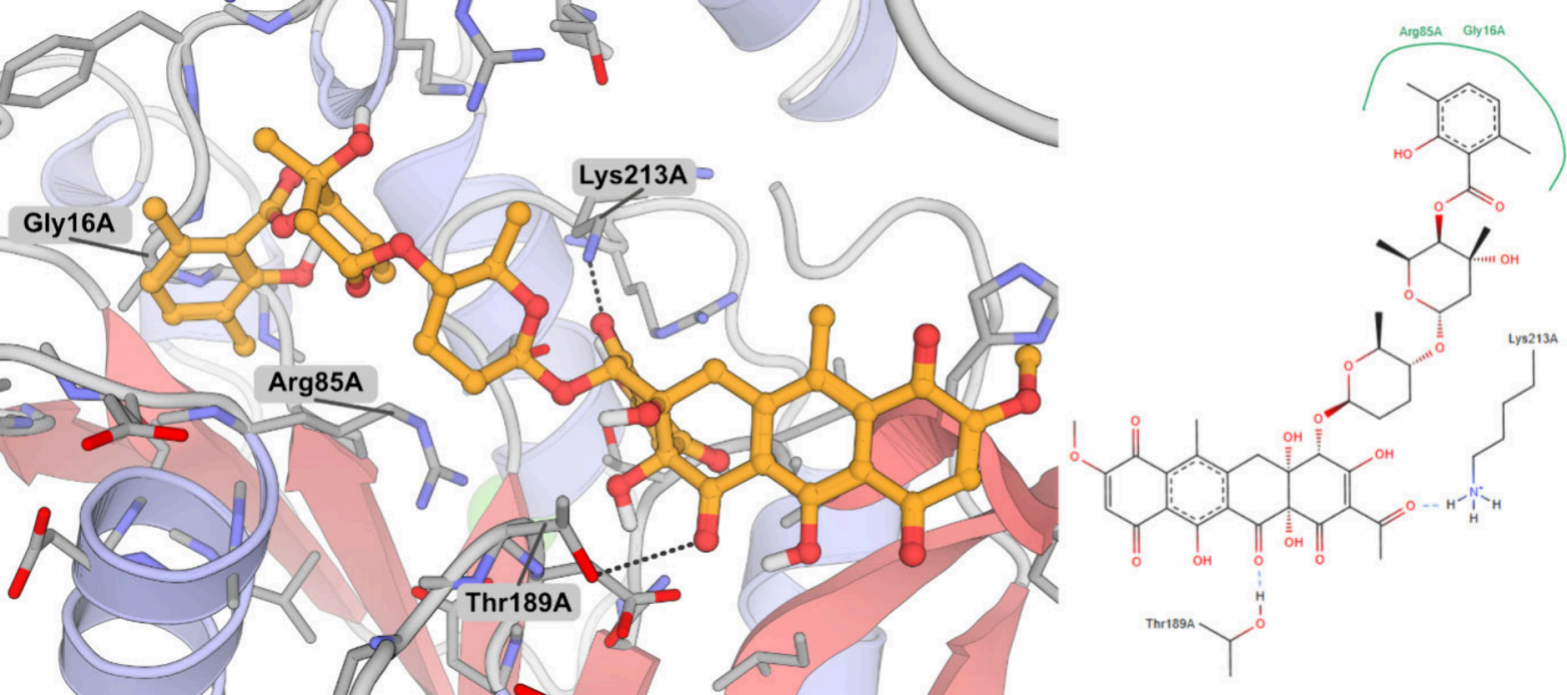
(left) 3D representation of the predicted binding mode of polyketomycin to *Escherichia coli* IspD. Polyketomycin is shown in orange. Interacting residues are labeled, and hydrogen bond interactions are shown as black, dashed lines. (right) 2D interaction diagram of polyketomycin with *Ec*IspD.

## Conclusions

Antimicrobial drug discovery in “beyond rule of five” chemical space holds great promise. NPs are a promising source for discovering novel skeletons with high structural diversity and various bioactivities that can be used directly or as starting points that undergo cycles of optimization to reach optimum drug-like properties. Nevertheless, hurdles for NP drug discovery are accessibility, sustainable supply, and IP constraints. It is also important to point out that the decline in the number of large companies actively engaged in NP research has played a key role. Here, we overcome most of these drawbacks by using the DZIF (German Center for Infection Research) NP libraries for *Mt*DXPS and *Ec*IspD enzymes along with other NP libraries for *Ec*IspD. Our screening was initiated with a hit-picking workflow based on an initial single-point screening assay of the NP libraries, followed by a counter-screening assay to eliminate potential false positives. Then, we moved forward by determining the IC_50_ values of the pure compounds and by checking the selectivity assays against human off-targets. We also evaluated the activity in human pathogen bacteria of the selected hits. Overall, our screening carried out against *Mt*DXPS and *Ec*IspD, the first and third enzymes of the MEP pathway, respectively, identified maracen A and polyketomycin as most promising hits in terms of on-target potency and selectivity. We also moved forward and studied their putative binding mode through docking studies. Yet further studies including cocrystallization with the corresponding enzyme would help to confirm their binding mode and guide future optimization and inhibitor development. Although the general need to optimize key pharmacological properties of such hits remains unquestioned and is beyond the scope of this study, our work adds a piece to the MEP inhibitors discovered so far. Given the current dearth of MEP-targeting drugs in clinical trials and the untapped opportunities to use MEP inhibitors in the development of anti-infectives, to the best of our knowledge, we are reporting the first NP screening against MEP pathway enzymes. Importantly, we are proving that our approach is feasible and robust and represents an alternative route to the hit-identification strategies reported so far for MEP pathway enzymes. In fact, to date, novel IspD and DXPS inhibitors have been identified mainly through the screening of *i*) a library of approved drugs (for *Ab*IspD),^24^ *ii*) a BASF proprietary library of about 100,000 compounds (for *At*IspD),^25^ *iii*) a “Malaria Box” library (for *Pf*IspD),^26,27^ and *iv*) using a target-directed dynamic combinatorial chemistry (for *dr*DXPS) approach.^28^ Taken together, our work provides a foundation to perform novel NP screening for the discovery of MEP pathway inhibitor and optimization of NP hits identified.

## Supplementary Material



## Notes

NP screenings and counter-screening assays (PDF)

## ¶

Alma Mater Studiorum–University of Bologna, Bologna 40126, Italy

## ●

Biozentrum, Basel University, Spitalstrasse 41, 385 4056 Basel, Switzerland

## ABBREVIATIONS

MEP: 2*C*-methyl-d-erythritol 4-phosphate
BAP: butylacetylphosphonate
IDP: isopentenyl diphosphate
DMADP: dimethylallyl diphosphate
DXPS: 1-deoxy-d-xylulose 5-phosphate synthase
d-GAP: d-glyceraldehyde 3-phosphate
PDH: pyruvate dehydrogenase
PK: pyruvate kinase
LDH: lactate dehydrogenase
