# Supplementary material for: Identification of Natural-Product Inhibitors of the 2C‑Methyl‑d‑erythritol 4‑Phosphate Pathway
Source: ACS Med Chem Lett. 2026 Mar 13;17(4):909–15. doi: 10.1021/acsmedchemlett.6c00054 (PMC13071607; doi:10.1021/acsmedchemlett.6c00054)
Supplement: Supplementary file 1 [file ml6c00054_si_001.pdf]

Supporting Information

Identification of natural product inhibitors of the MEP pathway

Eleonora Diamanti,[a,‡,†] *Alaa Alhayek,*[a,‡,†] *Antoine Lacour,*[a,b,c] *Mohammad Walid Shahrour,*[a,b,c] *Daan Willocx,*[a,b] *Boris Illarionov,*[d] *Markus Fischer,*[d] *Marc Stadler,*[e] *Norbert Reiling,*[f] *Jennifer Herrmann,*[a] *Rolf Müller,*[a,b] *and Anna K. H. Hirsch* [a,b,c*]

[a] Helmholtz Institute for Pharmaceutical Research (HIPS)-Helmholtz Centre for Infection Research (HZI), 66123 Saarbrücken, Germany.

[b] Saarland University, Department of Pharmacy, Campus Building E8.1, 66123 Saarbrücken, Germany.

[c] PharmaScienceHub, Campus, 66123 Saarbrücken, Germany.

[d] Hamburg School of Food Science, Institute of Food Chemistry, Grindelallee 117, 20146 Hamburg, Germany.

[e] Department Microbial Drugs, Helmholtz Centre for Infection Research (HZI), 38124 Braunschweig, Germany.

[f] Research Center Borstel, D-23845 Borstel, Germany.

* Prof. Anna K.H. Hirsch

anna.hirsch@helmholtz-hips.de

Table of Contents

1.0 *Mt*DXPS screening 3
1.1 Natural product library 3
2.0 *Mt*DXPS counter-screen assay 4
3.0 IC_{50} evaluation of *Mt*DXPS 5
4.0 Mammalian PDH inhibition assay 5
5.0 Activity of Maracen A on other bacterial MEP enzymes and on mammalian off-targets 6
6.0 Mode of inhibition study 6
6.1 Determination of steady-state kinetic parameters 6
7.0 *Escherichia coli* IspD photometric enzyme activity assay 7
7.1 IC_{50} curves of *Ec*IspD inhibitors 9
8.0 Molecular docking 12
8.1 Maracen A 12
8.2 Polyketomycin 12
References 12

1.0*Mt*DXPS screening

1.1 Natural product library

The screening experiments were performed as reported before with some modifications.[1] The assay was prepared using two buffers: buffer A2 and B2. To screen the library, a volume of 10 µL of buffer B2 (200 mM HEPES pH 8, 0.1% (*v/v*) TRITON X-100, 0.6 mM D-GAP and 0.2 mM sodium pyruvate) and 50 µM or 50 µg/mL of natural product or crude extract, respectively, was combined in a black 384-well microtiter plate (Greiner BioOne, Kremsmünster, Austria). To start the enzymatic reaction, 10 µL of buffer A2 (200 mM HEPES pH 8, 0.1% (*v/v*) TRITON X-100, 0.6 µM ThDP, 2 mM DTT, 2 mM $MgCl_2$, 0.1 mM NADPH, 3 µM IspC and 0.1 µM *Mt*DXPS) was added. We then centrifuged the plate for 1 min at 2000 rpm and 25 °C, after which the fluorescence intensity was monitored using a CLARIOstar plate reader (BMG Labtech, Ortenberg, Germany) for 40 min at RT. The percentage of inhibition was calculated relative to negative control (*i.e.*, no compound). Results are depicted in Figure S1.

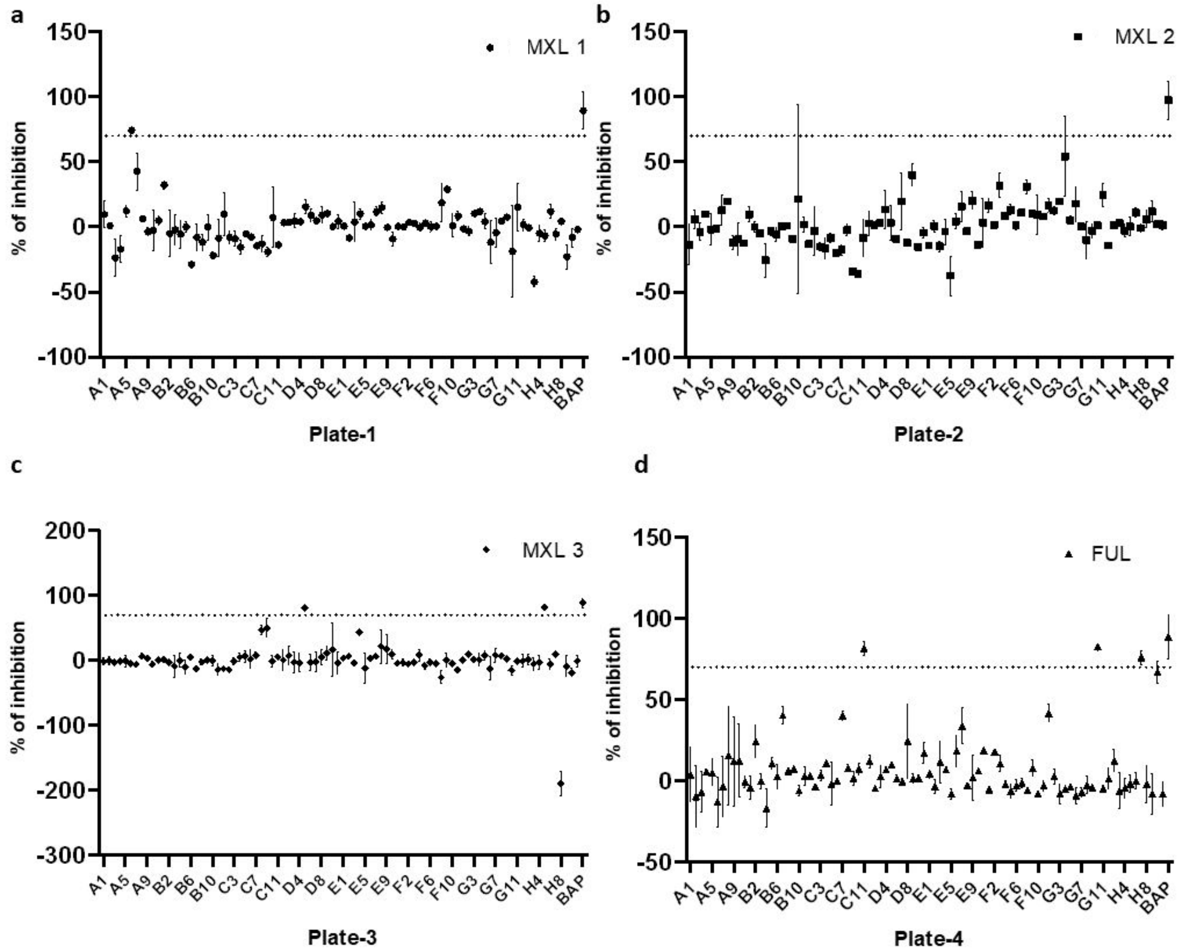

Figure S1. Depiction of percentage of inhibition of myxobacterial and fungal natural product on *Mt*DXPS. The dots of the scatter plot represent average data from at least two independent experiments, cut-off for the screening is 70% inhibition at 50 µM. BAP: Butylacetylphosphonate, *Mt*DXPS: *Mycobacterium tuberculosis* 1-Deoxy-D-Xylulose 5-Phosphate Synthase. MXL=myxobacterial NPs; FUL=fungal NPs.

Table S1. Activity of pure myxobacterial and fungal NP hits listed in alphabetical order on *Mt*DXPS[a]

	Compound	Origin/plate	% of inhibition on *Mt*DXPS at 50 µM
1	Cohaerin E	Fungi/FUL	40 ± 0
2	Cohaerin F	Fungi/FUL	40 ± 1
3	Glidobactin A	Myxo/MXL2	54 ± 1
4	Hexadecenoic acid	Myxo/MXL2	40 ± 0
5	Hyafurone 9 (B)	Myxo/MXL3	50 ± 2
6	Hymeron/ Hyafurone E	Myxo/MXL3	44 ± 1
7	Laetiporin A	Fungi/FUL	42 ± 2
8	Lysocin	*Lysobacter* spp	47 ± 3
9	Maracen A	Myxo/MXL1	74 ± 2
10	Obionin A	Fungi/FUL	42 ± 3
11	Obionin C	Fungi/FUL	76 ± 1
12	Sulfangolid C	Myxo/MXL1	42 ± 1
13	Sulfangolid A	Myxo/MXL3	81 ± 1
14	Sorangiolid A	Myxo/MXL3	82 ± 2
15	Truncaton A	Fungi/FUL	82 ± 1
16	Truncaton C	Fungi/FUL	67 ± 2

[a]Means and SD of at least two independent experiments are shown.

2.0 *Mt*DXPS counter-screen assay

To recognize the false-positive hits associated with the assay conditions, the *in vitro* inhibition assay was conducted as described in section 1.0. Buffer A2 (without *Mt*DXPS) and B2 were mixed with the hit compounds. The change in the fluorescence signal of NADPH was investigated with and without compounds. The compounds that demonstrated a drop in the NAHPD fluorescence signal over time were considered as false-positive hits.

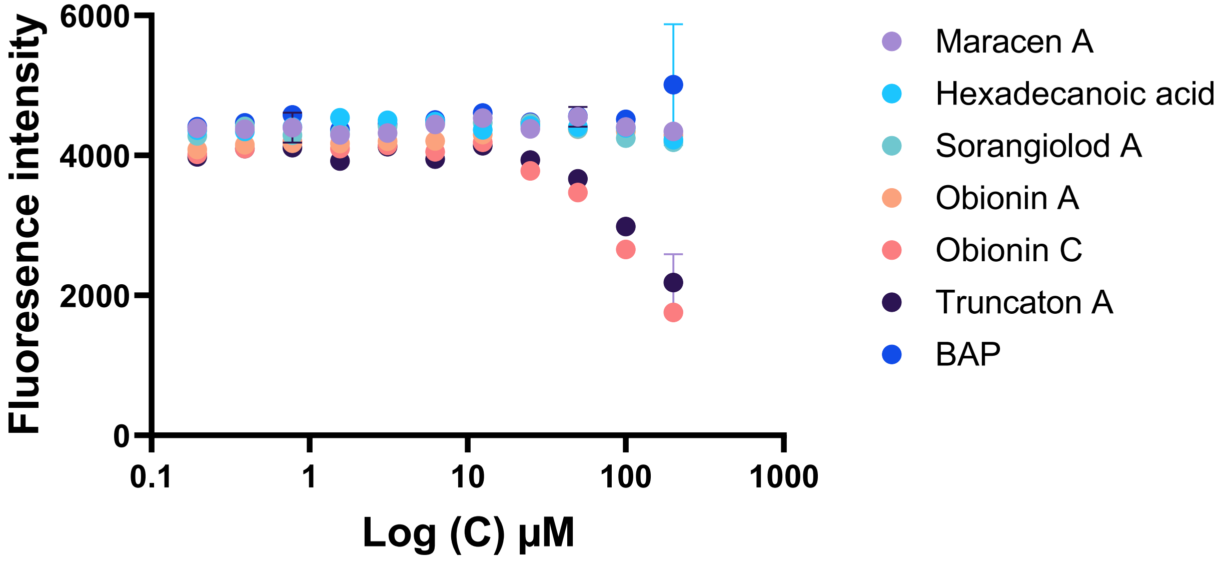

Figure S2. Representation of the false compound signals quenched NADPH fluorescence.
Means and SD of at least two independent experiments are shown. BAP: Butylacetylphosphonate, serves as a negative control.

3.0 IC_{50} evaluation of *Mt*DXPS

The assay was prepared as previously reported using two buffers; buffer A2 and B2.[1] To screen the library, a volume of 10 µL of buffer B2 (200 mM HEPES pH 8, 0.1% (*v/v*) TRITON X-100, 0.6 mM D-GAP and 0.2 mM sodium pyruvate) and 50 µM or 50 µg/mL of natural product or crude extract, respectively, was combined in a black 384-well microtiter plate (Greiner BioOne, Kremsmünster, Austria). To start the enzymatic reaction, 10 µL of buffer A2 (200 mM HEPES pH 8, 0.1% (*v/v*) TRITON X-100, 0.6 µM ThDP, 2 mM DTT, 2 mM $MgCl_2$, 0.1 mM NADPH, 3 µM IspC and 0.1 µM *Mt*DXPS) was added. We then centrifuged the plate for 1 min at 2000 rpm and 25 °C, after which the fluorescence intensity was monitored using a CLARIOstar plate reader (BMG Labtech, Ortenberg, Germany) for 40 min at RT.

a

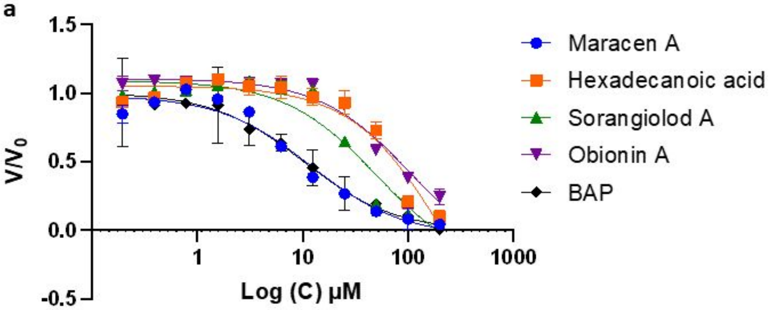

b

Compound	IC_{50} (µM) mtDXPS
Maracen A	10.3 ± 0.02
Hexadecanoic acid	72.2 ± 6.4
Sorangiolid A	21.2 ± 0.2
Obionin A	55.2 ± 3.3
BAP	10.3 ± 0.5

Figure S3. Calculated IC_{50} values of each hit against *Mt*DXPS.

4.0 Mammalian PDH inhibition assay

The assay was carried out as previously described.[2] Buffer A3 (100 mM HEPES pH 8, 0.1% (*v/v*) TRITON X-100, 1 mg/mL BSA, 1 mM DTT, 0.325 µM ThDP, 1 mM $MgCl_2$, 0.5 mM NAD^+, 2 mM cysteine HCl, 0.01 U/mL PDH from porcine heart (purchased from Sigma Aldrich) and buffer B3 (100 mM HEPES pH 8, 0.1% (*v/v*) TRITON X-100 with 0.15 mM coenzyme A and 0.15 mM sodium pyruvate) were prepared. A concentration of 100 µM compound and 30 µL of buffer A3 were combined in a 384-transparent microtiter plate (Greiner BioOne, Kremsmünster, Austria) and incubated for 10 min at 30 °C. To initiate the enzyme-activity reaction, we added 30 µL of buffer B3 and the absorbance of NADH was monitored with a PHERAstar plate reader (BMG Labtech, Ortenberg, Germany) at wavelength of 340 nm for 1 h at 30 °C. Percent of inhibition was calculated related to the negative control (no compound). Fluoropyruvate was included as a reference inhibitor.

a

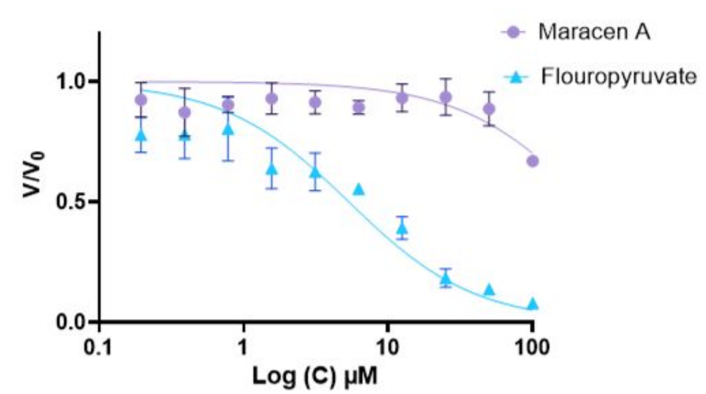

b

Compound	IC_{50} (μM) PDH
Maracen A	> 100
Flouropyruvate	5.3 ± 3.2

Figure S4. Dose-response curve of maracen A on mammalian PDH enzyme. Fluoropyruvate was used as a positive control reference inhibitor of pyruvate dehydrogenase (PDH).

5.0 Activity of Maracen A on other bacterial MEP enzymes and on mammalian off-targets

The activity of natural product hits on *Plasmodium falciparum* IspD, *Escherichia coli* IspD and IspE, and mammalian PK/LDH was tested following the previously reported procedures.

Table S2. IC_{50} values of Maracen A against other MEP enzymes

Compound	IC_{50} (μM)[a]				
	PDH	PK/ LDH	*Pf*IspD	*Ec*IspD	*Ec*IspE
Maracen A	>100	>500	26 ± 7	>500	>500

[a]Means and SD of at least two independent experiments

6.0 Mode of inhibition study

6.1 Determination of steady-state kinetic parameters

The kinetic parameters of *Mt*DXPS substrates (*i.e.,* D-GAP and sodium pyruvate) and co-factor (*i.e.,* ThDP) were determined with a DXPS-IspC coupled assay. The kinetic parameters of D-GAP were calculated by titrating 10 mM D-GAP on 2 mM sodium pyruvate. While sodium pyruvate and ThDP parameters were defined by titrating 3 mM sodium pyruvate on 4 mM D-GAP and 2.9 mM ThDP on 2 mM sodium pyruvate and 4 mM D-GAP, respectively. The titration step was performed with a 1.5x dilution factor in a 384-well microtiter plate (Greiner BioOne, Kremsmünster, Austria). In all cases, the enzymatic reaction was started by adding buffer A1 which contains 200 mM HEPES pH 8, 4 mM DTT, 1 mM Mg^{2+}, 0.1 mM NADPH, 3 μM IspC and 0.1 μM *Mt*DXS. The plate was then centrifuged for 1 min at 2000 rpm and 25 °C. The fluorescence intensity of NADPH consumption,

which is directly related to DXP formation, was monitored over 30 min at RT using a CLARIOstar plate reader (BMG Labtech, Ortenberg, Germany). The fluorescence was measured at an excitation wavelength of 340 ± 15 nm and an emission wavelength of 415 ± 20 nm. The data analysis was performed using GraphPad Prism v9. The steady-state kinetic parameters (k_{cat}, K_M) were determined by non-linear regression and with Michaelis-Menten equation (Equation 1).

**Equation 1 Michaelis-Menten steady-state kinetic calculation**<br>$V_o = V_{max} [C][D] / (K_M^{pyruvate}[D]+K_M^{D\text{-}GAP}[C]+[C][D])$	
V_o	The initial velocity of DXP formation
V_{max}	The maximum velocity of DXP formation obtained when the enzyme is saturated by one of the substrate
K_M	The Michaelis constant and its value equal the concentration of the substrate at the half of V_{max}
C	Pyruvate concentration
D	D-GAP concentration
K_M pyruvate	The Michaelis constant of pyruvate at the saturating of the second substrate
K_M D-GAP	The Michaelis constant of D-GAP at the saturating of the second substrate

Table S3: Steady-state kinetic parameters of *Mt*DXPS substrates and co-factor[a]

**Kinetic parameter**	**Pyruvate**	**D-GAP**	**ThDP**
K_M (mM)	0.11 ± 0.02	0.36 ± 0.03	0.0003 ± 0.05
k_{cat} (min^{-1})	15 ± 0.04	14 ± 0.04	16 ± 0.3
k_{cat}/K_M (min^{-1}. mM^{-1})	136.36	38.88	53,333.33

[a]Means and SD of at least two independent experiments.

6.2 Competition study between Maracen A and ThDP

The mode of inhibition was determined using a modified version of a previously described protocol.[3] To study the competition of Maracen A with ThDP. The cofactor was titrated in the 0.3–50 µM range. Four ThDP concentrations were used: 0.3 µM, 3 µM, 30 µM, and 50 µM covering the range from 1x K_MThDP to 166x K_MThDP. The serially diluted inhibitor was added to buffer A2, and the enzymatic reaction was started with the addition of buffer B2. The rest of the procedure followed the IC_{50} determination method (Section 3.0).

7.0 *Escherichia coli* IspD photometric enzyme activity assay

Assays were conducted in 384-well plates (Corning) with a transparent flat bottom. Assay buffer A (total volume, 30 µL) contained 200 mM Tris hydrochloride, pH 7.6, 20 mM KCl, 10 mM $MgCl_2$, 10 mM DTT, 1.2 mM NADH, 2 mM ATP, 2 mM phosphoenolpyruvic acid, 0.2 U of pyruvate kinase (PK), 0.2 U of lactate dehydrogenase (LDH), 0.2 U (1.8 µM) IspE from *E. coli* (*Ec*IspE) and 0.004 U (0.050 µM) of IspD protein from *Escherichia coli* (*Ec*IspD). Dilution series were performed with dilution step 1:2 and approximately covered the concentration range of 200 µM to 0.01 µM of tested compounds. The reaction was started by addition of the assay buffer B that contained 200 mM Tris hydrochloride, pH 7.6, 1 mM MEP and 1 mM CTP.

The impact of the tested compounds on auxiliary enzymes in the photometric activity inhibition assay was tested as follows. Test buffer A (total volume, 30 µL) contained 200 mM Tris hydrochloride, pH 7.6, 20 mM KCl, 10 mM $MgCl_2$, 10 mM DTT, 1.2 mM NADH, 2 mM ATP, 2 mM phosphoenolpyruvic acid, 0.2 U of pyruvate kinase

(PK), 0.2 U of lactate dehydrogenase (LDH) and 0.004 U (0.02 µM) *Ec*IspE. Dilution series were performed with dilution step 1:2 and approximately covered the concentration range of 200 µM to 0.01 µM of tested compounds. The reaction was started by addition of the assay buffer B that contained 200 mM Tris hydrochloride, pH 7.6, 1 mM CDP-ME. The reactions were monitored photometrically (room temperature) at 340 nm in a plate reader (SpectraMax5, Molecular Dynamics). Initial rate values were evaluated with a nonlinear regression method using the program Dynafit.[5] MEP and CDP-ME were used as starting substrate in the IspD and IspE assay, respectively, and were synthesised and purified as described earlier.[6]

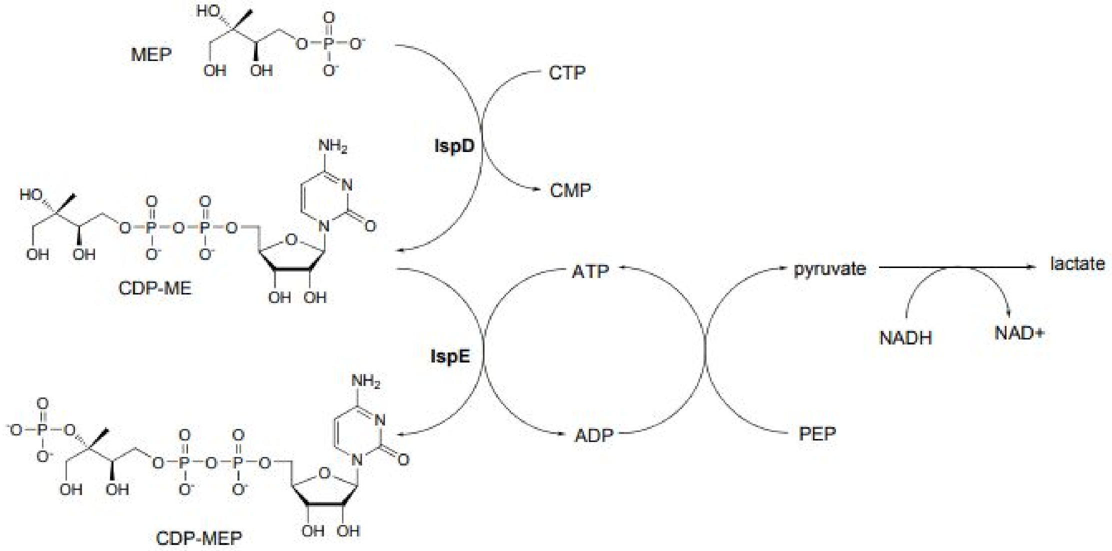

Scheme S1. Photometrical IspD assay.

MEP, 2-*C*-methyl-D-erythritol 4-phosphate; CDP-ME, 4- diphosphocytidyl-2-*C*-methyl- D -erythritol; CDP-MEP, 4-diphosphocytidyl-2-*C*-methyl-D-erythritol 2-phosphate; IspD, 2-*C*-methyl-D-erythritol 4-phosphate cytidylyltransferase; IspE, 4-diphosphocytidyl-2-*C*-methyl- D-erythritol kinase.

Table S4. Percentage of inhibition of the NP library listed in alphabetical order against *Ec*IspD.

	**Compound**	**Origin/plate**	**% of inhibition on *Ec*IspD at 25 µM**
1	Averantin	BioVL1-2	30.4
2	Gamma-Rubromycin	BioVL1-2	33
3	Hormaomycin	BioVL1-2	32.3
4	Isochaetochromin B1	BioVL1-2	77.8
5	Kendomycin	BioVL1-2	32.2
6	Obionin C	Fungi/FUL	54.1
7	Obionin A	Fungi/FUL	25.6
8	Palmarumycin C3-5.8-quinone	BioVL1-2	60.0
9	Pentabromopseudilin	BioVL1-2	56.3
10	Polyketomycin	BioVL1-2	74.8
11	Simocyclinone D4	BioVL1-2	55.1
12	Skyrin	BioVL1-2	62.5
13	Truncaton A	Fungi/FUL	57.8
14	Truncaton C	Fungi/FUL	58.5

15	Violacein	BioVL1-2	43.5

7.1 IC_{50} curves of *Ec*IspD inhibitors

Skyrin

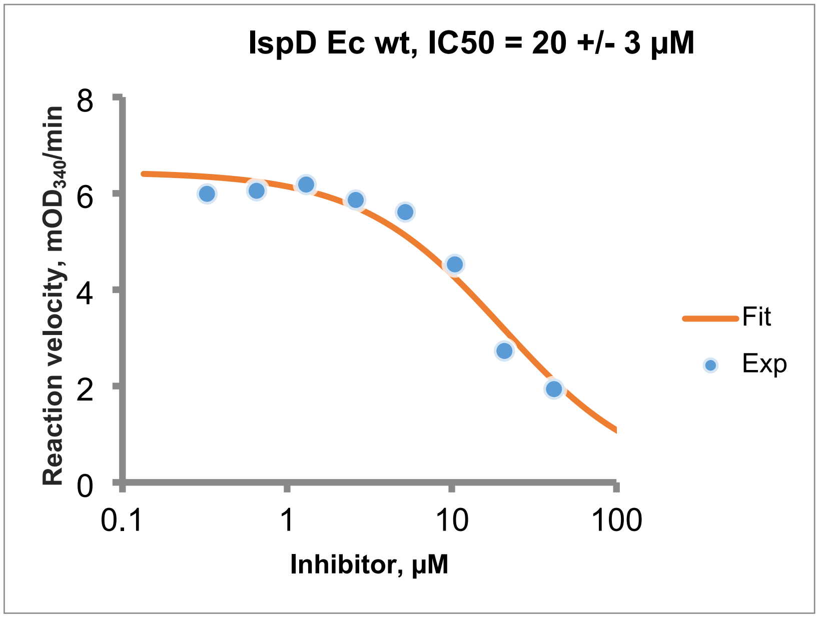

Simocyclinone D4

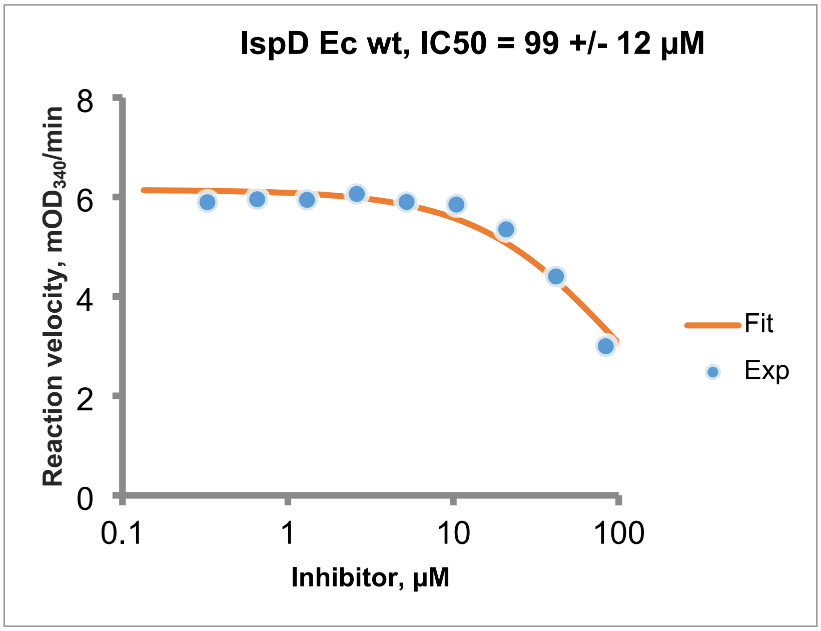

Palmarumycin

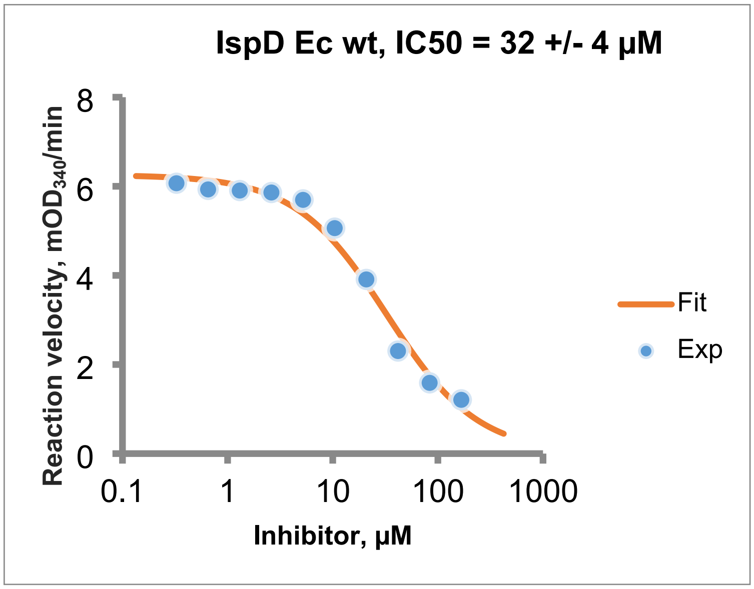

Polyketomycin

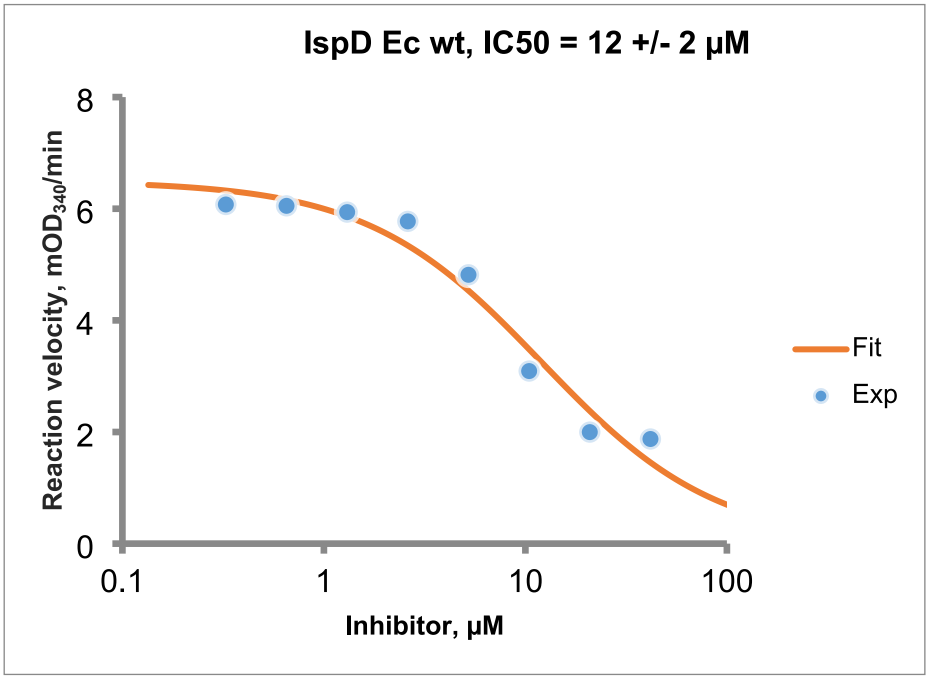

Pentabromopseudilin

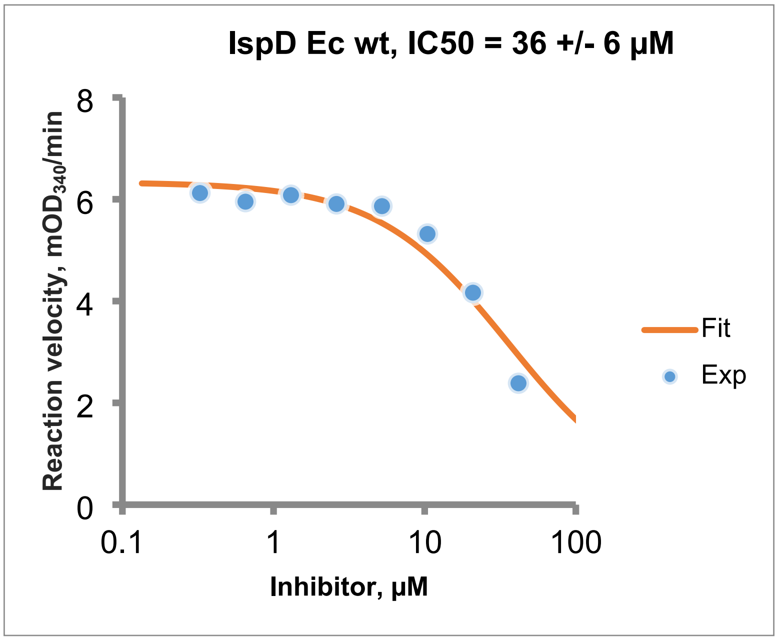

Table S5. IC_{50} data of NP compounds.

	Skyrin	Palmarumycin	Polyketomycin	Pentabromopseudilin
**Enzyme**	**IC_{50} (µM)[a]**			
*Ec*IspD	20±3	32±4	12±2	36±6
*Ec*IspE	128±93	>500	>500	>500
PK-LDH	73±10	>500	>500	30±4

[a]Means and SD of at least two independent experiments.

Table S6. Antibacterial and cytotoxic properties of *Ec*IspD hits.

	Skyrin	Palmarumycin	Polyketomycin	Pentabromopseudilin
**Strain**	**MIC [µg/mL]**			
Escherichia coli DSM-1116	> 128	> 128	> 128	16
Escherichia coli BW25113 (WT)	> 128	> 128	> 128	32
Escherichia coli BW25113 (WT) + 3 µg/mL PMBN	> 128	64	4	0.25
Escherichia coli Δ*acrB* (JW0451-2)	> 128	> 128	> 128	16
Escherichia coli Δ*tolC*	> 128	> 128	> 128	0.06
Klebsiella pneumoniae DSM-30104	> 128	> 128	> 128	16
Enterococcus faecium DSM-20477	> 128	> 128	0.0625	2
Staphylococcus aureus Newman	2	16	0.0625	0.0625
**Cell line**	**IC_{50} [µg/mL]**			

KB3.1	51.8	0.7	19.2	4.7

8.0 Molecular docking

8.1 Maracen A

*Mt*DXPS crystal structure (PDB code: 7A9H[7]) was downloaded and visualized in PyMOL.[8] Chain B and ThDP in the active site were removed. Given the presence of water molecules coordinated by the Magnesium ion (Mg^{2+}) in the active site, water molecules HOH857 and HOH959 were preserved and all other water molecules were removed. The protonation state of the protein was predicted using Protoss.[9] The ligand structure was created in ChemDraw, and a three-dimensional conformer was generated and minimized using RDkit.[10] SeeSAR 13.1 was used to generate the docking poses as it handled coordination to Mg better in our prior experience. The protein and ligand were loaded into the SeeSAR interface. The binding was defined using the Binding Site mode within SeeSAR[11], using the cognate ThDP as a reference ligand. Poses were generated using default clash and conformation parameters, with the number of generated poses set to 100. The top pose was determined using the HYDE score integrated into SeeSAR. The protein-ligand interaction-diagram was created using PoseEdit.[12]

8.2 Polyketomycin

The *Ec*ISPD crystal structure (PDB code: 1I52[13]) was downloaded and visualized in PyMOL.[8] CTP and Water molecules were removed manually. The protonation state of the protein was predicted using Protoss.[9] The structure of Polyketomycin was downloaded from CHEMBL, and a three-dimensional conformer was generated and minimized using RDkit. The molecular docking runs were performed using GNINA[14] with "--exhaustiveness" set to 64. A seed value of 0 was used to ensure reproducibility. The number of binding modes was set to 100 and the generated poses were scored using the default "--cnn_scoring rescore" option. Top scoring poses were determined using the CNNscore scoring function. GNINA's "--*autobox_ligand*" option was used for defining the grid box with CDP-ME (coordinates taken from PDB code: 1INI) set as the reference ligand. The "--autobox_extend" option was applied in order to ensure that the dimensions of the formed grid box cover the generated Polyketomycin poses. The protein-ligand interaction-diagram was created using PoseEdit.[12]

References

(1) Masini, T.; Pilger, J.; Kroezen, B.S.; Illarionov, B.; Lottmann, P.; Fischer, M.; Griesinger, C.; Hirsch, A. K.H. De Novo fragment-based design of inhibitors of DXS guided by spin-diffusion-based NMR spectroscopy. *Chem. Sci.* **2014**, *5* (9), 3543–3551.

(2) Morris, F.; Vierling, R.; Boucher, L.; Bosch, J.; Meyers, F. C. L. DXP synthase-catalyzed c-n bond formation: nitroso substrate specificity studies guide selective inhibitor design. *ChemBioChem* **2013**, *14*

(11), 1309–1315.

(3) Marcozzi, A.; Masini, T.; Zhu, D.; Pesce, D.; Illarionov, B.; Fischer, M.; Herrmann, A.; Hirsch, A.K.H. Phage display on the anti-infective target 1-Deoxy-D-Xylulose-5-Phosphate synthase leads to an acceptor–substrate competitive peptidic inhibitor. *ChemBioChem* **2018**, *19* (1), 58–65.

(4) Jumde, R.P.; Guardigni, M.; Gierse, R. M.; Alhayek, A.; Zhu, D.; Hamid, Z.; Johannsen, S.; Elgaher, W. A. M.; Neusens, P. J.; Nehls, C.; Haupenthal, J.; Reiling, N.; Hirsch, A.K.H. Hit-optimization using target-directed dynamic combinatorial chemistry: development of inhibitors of the anti-infective target 1-Deoxy-D-Xylulose-5-Phosphate Synthase. *Chem. Sci.* **2021**, *12* (22), 7775–7785.

(5) Kuzmič, P. Program DYNAFIT for the analysis of enzyme kinetic data: application to HIV proteinase. *Anal. Biochem.* **1996**, *237* (2), 260–273.

(6) Honold, A.; Lettl, C.; Schindele, F.; Illarionov, B.; Haas, R.; Witschel, M.; Bacher, A.; Fischer, M. Inhibitors of the bifunctional 2-*C*-methyl-D-erythritol 4-phosphate cytidylyl transferase/2-C-methyl-D-erythritol-2,4-cyclopyrophosphate synthase (IspDF) of Helicobacter Pylori. *Helv. Chim. Acta* **2019**, *102* (3).

(7) Gierse, R. M.; Oerlemans, R.; Reddem, E.R.; Gawriljuk, V.O.; Alhayek, A.; Baitinger, D.; Jakobi, H.; Laber, B.; Lange, G.; Hirsch, A.K.H.; Groves, M. R. First crystal structures of 1-deoxy-D-Xylulose 5-phosphate synthase (DXPS) from Mycobacterium Tuberculosis indicate a distinct mechanism of intermediate stabilization. *Sci. Rep.* **2022**, *12* (1), 1–13.

(8) The PyMOL Molecular Graphics System. Schrödinger, LLC 2015.

(9) Bietz, S.; Urbaczek, S.; Schulz, B.; Rarey, M. Protoss: A holistic approach to predict tautomers and protonation states in protein-ligand complexes. *J. Cheminform.* **2014**, *6* (1), 1–12.

(10) Landrum, G.; Tosco, P.; Kelley, B.; Rodriguez, R.; Cosgrove, D.; Vianello, R.; Sriniker; Gedeck, P.; Jones, G.; Schneider, N.; Kawashima, E.; Nealschneider, D.; Dalke, A.; Swain, M.; Cole, B.; Turk, S.; Savelev, A.; Vaucher, A.; Wójcikowski, M.; Take, I.; Scalfani, V. F.; Walker, R.; Probst, D.; Ujihara, K.; Pahl, A.; Godin, G.; Lehtivarjo, J.; Bérenger, F.; Bisson, J. Rdk. Rdkit/Rdkit. 2024.

(11) SeeSAR V13.1. www.biosolveit.de/SeeSAR .

(12) Diedrich, K.; Krause, B.; Berg, O.; Rarey, M. PoseEdit: enhanced ligand binding mode communication by interactive 2D diagrams. *J. Comput. Aided. Mol. Des.* **2023**, *37* (10), 491–503.

(13) Richard, S.B.; Bowman, M.E.; Kwiatkowski, W.; Kang, I.; Chow, C.; Lillo, A. M.; Cane, D.E.; Noel, J. P. Structure of 4-diphosphocytidyl-2-*C*-methylerythritol synthetase involved in mevalonate-independent isoprenoid biosynthesis. *Nat. Struct. Biol.* **2001**, *8* (7), 641–648.

(14) McNutt, A. T.; Francoeur, P.; Aggarwal, R.; Masuda, T.; Meli, R.; Ragoza, M.; Sunseri, J.; Koes, D. R. GNINA 1.0: Molecular docking with deep learning. *J. Cheminform.* **2021**, *13* (1), 1–20.
